# A novel nucleic acid extraction method from aromatic herbs and dried herbal powders using cow skim milk

**DOI:** 10.1038/s41598-020-68467-4

**Published:** 2020-07-13

**Authors:** Sunil Kumar Verma, Nabanita Biswas

**Affiliations:** S212, CSIR – Centre for Cellular and Molecular Biology, Uppal Road, Hyderabad, 500 007 India

**Keywords:** Biological techniques, Plant sciences

## Abstract

Authenticity of dried aromatic herbs and herbal powders for the ASU (ayurvedic, siddha, unani) drug formulations is a key of their clinical success. The DNA based authentication is an answer; however, extraction of PCR quality DNA from such material is often problematic due to the presence of various co-extracted PCR inhibitors. Here, we report a novel DNA isolation and purification method utilizing cow skim milk that successfully yields PCR quality DNA from the aromatic herbs and dried herbal powders. The improved method presented in this study could be used as an alternative to successfully extract PCR quality DNA from such plant materials. Further, we present a set of robust *matK* primers which could be used as plant barcoding resource in future studies.

## Introduction

The medicinal plant based formulations have long been used for the treatment of various human ailments since ancient times till modern days^1,2^. The unsustainable use of these plants has created a negative pressure on their biodiversity and availability; which also leads to unethical malpractices in the current times by the practitioners and traders of the medicinal plants^3–7^. During recent years, there have also been reports pertaining to the toxicity of various plant based formulations. Such complains, however, are also being attributed to a larger extent with the unethical malpractices such as mislabeling, fraudulently replacement as well as unintentional negligence^8^. Since most of the times the real identity of the plant remains spurious; therefore, unintended plant formulation is given to the patient, leading to toxicity^8^. Thus, the correct identification and authentication of medicinal plants for their safe use is the need of the hour and seeks global attention.

DNA barcoding^9^ has emerged as a modern reliable tool for the identification and authentication of individual medicinal and herbal plants at molecular level^7,10,11^. This however, requires extraction of PCR quality DNA from such material. Extraction of DNA from herbal and aromatic plants is often problematic, because these plants contain high levels of secondary metabolites including lipids, phenolic compounds, and viscous polysaccharides that can interfere with downstream molecular applications. The problem becomes even worse if the material under investigation is dried herb or herbal powder, since the quantity and purity of DNA often recovered from such material is not up to the mark, leading to the failure of downstream molecular applications^12^. Thus, the DNA isolation from such herbal materials so far heavily depends on expensive commercial kits^3,12–15^.

Here, we have developed a novel method for the extraction of PCR quality DNA from aromatic herbs and dried herbal powders, without the use of any expensive commercial DNA extraction kit. Our method is modified from the cetyltrimethylammonium bromide (CTAB) method^16^ and the key novel step in this method is the use of cow skim milk (0.1% fat) during the CTAB lysis of dried herbs and aromatic plants. Skim milk possibly acts by adsorbing the DNA and competing with other adsorption competitors and impurities present in the crude lysates^17^. In the second stage of our procedure, the skim milk adsorbed DNA is purified and separated from co-extracted impurities using routine phenol:chloroform extraction^18^.

## Material and methods

### The plant material

**Figure 1:**
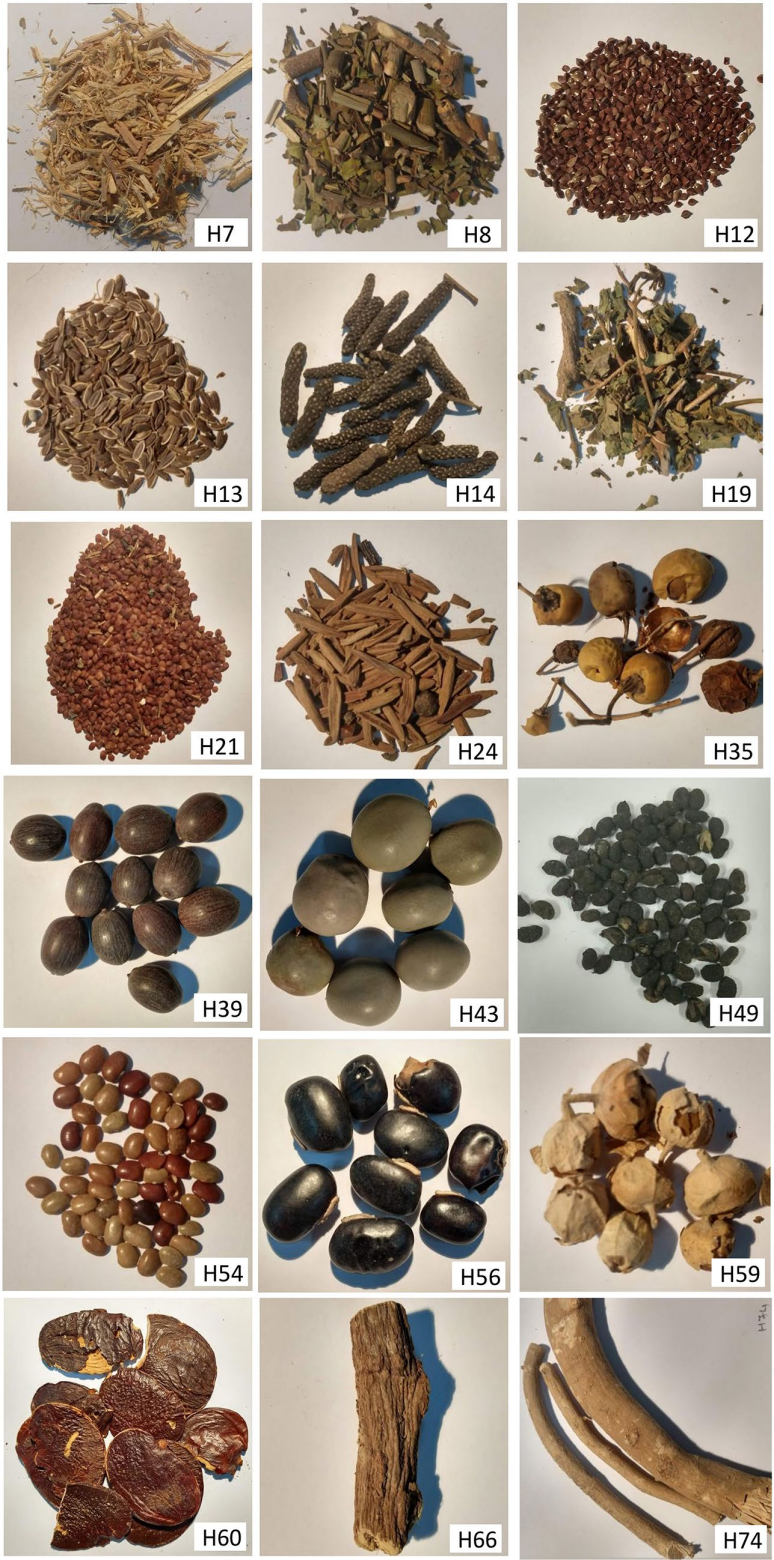
Dried herbs included in this study. Refer to Table 2 for the details.

Various dried herbs, which often include the dried seed, stem part, flowers or any other part of various herbal plants, are being sold in the online marketplace in India. Since these herbs are dried, these are often stored in shops and other marketplace at room temperature. We ordered small quantities of a total of 18 randomly selected dried herbs from an online herbal marketplace (see Tables 1, 2, Fig. 1), which were delivered to us in individually sealed and labeled small packets by routine parcel at room temperature. Once arrived in the lab, a specific voucher code was given to each sample (see Table 2) and these were stored at room temperature until further processing. Further, the 35 fresh aromatic herbal plants (mostly the fresh leaves) were randomly picked up from countryside land near Hyderabad, India (Supplementary Fig. 1, Supplementary Table 3). The five ayurvedic powders included in this study were personally obtained by SKV from a traditional practitioner of Ayurveda (see Supplementary Fig. 2, Supplementary Table 4).

**Table 1:** Details of the 12 randomly selected dried herbs (Photographs of each of these herbs are provided in Fig. 1) used for the standardization of the DNA extraction procedure developed in this study.

S.N	Herb code	Herb name (on the label)	DNA extraction (without milk)		DNA extraction (with milk)	
			Conc (ng/ul)	OD (260/280	Conc (ng/ul)	OD (260/280
1	H7	Dashmool Kwath—Dashmool Bharad	70.7	1.72	116.8	1.87
2	H8	Adulsa—*Justicia adhatoda*	194.6	1.83	318.6	1.79
3	H14(1)	Lendi Pipali—*Piper longum* seed	20.1	1.35	383.9	1.78
	H14 (2)	Lendi Pipali—*Piper longum* seed	144.1	1.71	N/A*	
4	H19	Gudmar—*Gymnema sylvestre*	1,216.2	2.04	556.6	1.79
5	H24	Indrajav—*Holarrhena pubescens*	638.0	1.39	358.6	1.80
6	H34 (1)	Gulvel—*Tinospora cordifolia*	157.4	2.07	30.3	1.80
	H34 (2)	Gulvel—*Tinospora cordifolia*	149.7	2.01	N/A*	
7	H35 (1)	Kantkari/Ringani—*Solanum surattense*	215.0	1.75	151.7	1.55
	H35 (2)	Kantkari/Ringani—*Solanum surattense*	178.3	1.76	N/A*	
8	H44	Vavding—*Embelia ribes*	519.7	2.03	2,560.9	1.84
9	H49	Bavchi—*Psoralea corylifolia*	3,423.5	1.95	848.0	1.91
10	H54	Kulathi—*Dolichos biflorus*	479.6	1.75	176.9	1.66
11	H59	Paneer Phul—*Withania coagulans*	185.2	1.65	321.8	1.59
12	H74	Miswak—*Salvadora persica*	117.2	2.03	313.9	1.80

*This set not processed with milk due to limited amount of available samples.

**Table 2:** Various dried herbs included in this study to validate the DNA extraction procedure and the success of downstream molecular analysis.

S.N	Sample code	Dried herb name as on label /voucher code	NCBI accession no.*	Highest bits	Query cover %	BLAST E value	% Nucleotide Similarity	NCBI accession no.**	Herb identity revealed as
1	H7	Dashmool Kwath^†^—Dashmool Bharad (CCMB:29–122:H7)	MN006741	1,321	100	0.0	100	MF694887.1	*Tribulus *sp.* CCMB H7*^1^
2	H8	Adulsa—*Justicia adhatoda* (CCMB:29–123:H8)	MN006742	1,317	100	0.0	99.86	MG947002.1	*Justicia adhatoda/Adhatoda vasica*^2^
3	H12	Beejband^††^—*Sida cordifolia* (CCMB:29–128:H12.1)	MN006743	1,327	100	0.0	100	KY952501.1	*Rumex *sp.* CCMB H12*
4	H13	Balantshepa/Dill Seed/Savaa Seed/*Anethum graveolens* Seed (CCMB:29–131:H13.2)	MN006744	1,267	100	0.0	100	MG946951.1	*Anethum graveolens*
5	H14	Lendi Pipali—*Piper longum* seed (CCMB:30–134:H14)	MN006745	1,360	100	0.0	99.73	MH287271.1	*Piper longum*^3^
6	H19	Gudmar—*Gymnema sylvestre* (CCMB:30–135:H19)	MN006746	1,391	100	0.0	100	KX911179.1	*Gymnema sylvestre*
7	H21	Lajvanti Beej^††^—*Mimosa pudica* Seed (CCMB:29–129:H21)	MN006747	1,303	100	0.0	99.17	GU135078.1	*Hygrophila polysperma*
8	H24	Indrajav—*Holarrhena pubescens* (CCMB:29–130:H24)	MN006748	1,371	100	0.0	99.87	EF456271.1	*Holarrhena pubescens*
9	H35	Kantkari/Ringani—*Solanum surattense* (CCMB:30–136:H35)	MN006749	1,291	100	0.0	100	MH085988.1	*Solanum *sp.* CCMB H35*
10	H39	Kamal Beej/ Kamal Gatta—*Lotus* Seed (CCMB:33–144:H39)	MN006750	1,295	100	0.0	100	LC438879.1	*Nelumbo *sp.* CCMB H39*
11	H43	Sagar Goti/Latakaranj—Molucca Bean/ *Caesalpinia bonduc* (CCMB:33–145:H43)	MN006751	1,365	100	0.0	99.87	LC080892.1	*Caesalpinia *sp.* CCMB H43*
12	H49	Bavchi—*Psoralea corylifolia* (CCMB:30–139:H49)	MN006752	1,387	100	0.0	100	MK069582.1	*Cullen corylifolium/Psoralea corylifolia*^4^
13	H54	Kulathi—*Dolichos biflorus* (CCMB:30–137:H54)	MN006753	1,376	100	0.0	100	EU717410.1	*Macrotyloma *sp.*/Dolichos biflorus*^5^
14	H56	Kavach Beej Black—*Mucuna pruriens* Seed (CCMB:33–142:H56)	MN006754	1,315	100	0.0	99.72	KF621103.1	*Mucuna cochinchinensis/Mucuna pruriens*^6^
15	H59	Paneer Phul—*Withania coagulans* (CCMB:30–133:H59)	MN006755	1,371	100	0.0	100	MG947039.1	*Withania *sp.* CCMB H59*
16	H60	Palas Beej—*Butea monosperma* Seed (CCMB:34–241:H60)	MN006756	970	100	0.0	100	KY628018.1	*Butea monosperma*^7^
17	H66	Jeshtmadh Kadi—*Glycyrrhiza uralensis* (CCMB:33–143:H66)	MN006757	1,295	100	0.0	100	MG736059.1	*Glycyrrhiza *sp.* CCMB H66*
18	H74	Miswak—*Salvadora persica* (CCMB:30–140:H74)	MN006758	1,352	99	0.0	100	MF694876.1	*Salvadora *sp.*/CCMB H74*

Photographs of each of these dried herbs are provided in Fig. 1.

*NCBI Accession Nos. of the novel sequences generated in this study.

**NCBI Accession No. of the best BLAST hit with the corresponding sequences in NCBI database.

^1^The highest BLAST score of the *matK* signature sequence obtained from this sample was with that of the signature sequence of *Tribulus sp*. NCBI accession no. MF694887.1 indicating its identity as that of the *Tribulus sp*. The identity of this specimen was confirmed by inclusion of reference samples for *Tribulus sp.* (Reference herb code WB35—Supplementary Fig. 3, Supplementary Table 5)*.* This also indicated that the taxonomic fidelity of the *matK* sequences generated in the current study could be considered as high.

^2,3,4,7^The identity of these specimen vouchers of dried herbs was also confirmed by inclusion of reference samples *for Adhatoda vasica/Justicia adhatoda*^*2*^ (synonyms)*, Piper longum*^*3*^*, Psoralea *sp./*Cullen* sp.^*4*^ (synonyms), and *Butea monosperma*^*7*^, respectively (Reference code CH73, CH10, SV3/J and WB4 respectively in Supplementary Fig. 3). The amplified *matK* sequences from these reference samples were comparable to that of the test herb (Supplementary Table 5).

^5^*Dolichos* and *Macrotyloma* genus are synonyms.

^6^*Mucuna pruriens* and *Mucuna cochinchinensis* are synonyms.

^†^According to standard text of Ayurveda, ‘Dashmool’ is the name given to 10 roots of certain plants that include Bilva root (*Aegle marmelos*), Agnimantha root (*Premna integrifolia*), Shyonaka root (*Oroxylum indicum*), Patala root (*Stereospermym suaveolens*), Kashmari root (*Gmelina arborea*), Bruhati root (*Solanum indicum*), Kantakari root (*Solanum xanthocarpum*), Shalaparni root (*Desmodium gangeticum*), Prushniparni root (*Uraria picta*), Gokshura root (*Tribulus terrestris*). The herb ‘Dashmool Bharad’ is sold in online marketplace without the clue of its scientific name. A search in Pubmed using keyword “Dashmool Bharad” also finds only one reference about it (*J Ayurveda Integr Med*, (2015), S26–S32. PubMed Central PMCID: PMC4456680), wherein, the in vitro evaluation of Anti-oxidant and Anti-inflammatory activities of Dashmool Bharad has been done. However, this reference also does not mention the scientific identify of ‘Dashmool Bharad’. Our study revealed that the ‘Dashmool Bharad’ is indeed the plant part of *Tribulus *sp*.*, one of the 10 components of ‘Dashmool’.

^††^Identity of these dried herbs was found to be spurious either due to mislabeling or fraudulently replacement, indicating the incidences of malpractices in online herbal marketplace.

**Figure 2:**
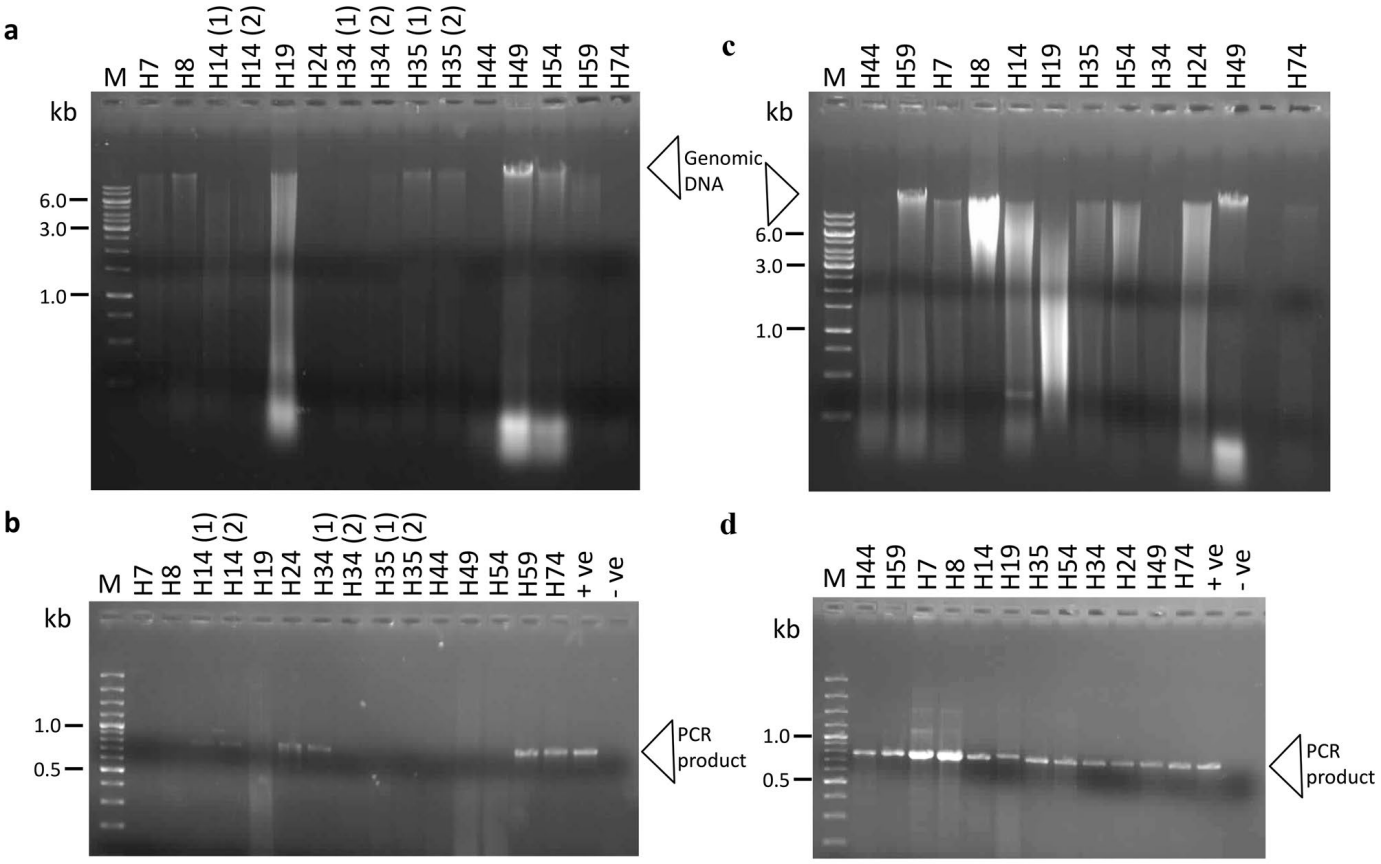
Agarose gel images showing the patterns of isolated DNA and amplified PCR products obtained from dried herbs included in this study. The DNA was isolated from 12 randomly selected dried herbs (Table 1) without (a) or with (c) addition of skim milk in the first step of DNA isolation as described in Methods section. The DNA obtained from both sets of experiments was subjected to PCR amplification using modified *matK* primers developed in this study (see material and methods section). The PCR band obtained from untreated samples are shown in panel ‘b’; and the PCR band obtained from skim milk treated samples are shown in panel ‘d’. The lanes marked as ‘+ve’ in panels ‘b’ and ‘d’ are the ‘positive’ control for PCR and the lanes marked as ‘–ve’ are the ‘internal negative control’ for PCR reactions. The molecular weight markers (Lane M) used are Thermo Scientific GeneRuler™ 1 kb DNA Ladder (panel ‘a’ and ‘c’) and Thermo Scientific GeneRuler™ 100 bp Plus DNA Ladder (panel ‘b’ and ‘d’). The uncropped, multiple original exposures of the full length agarose gels displayed here are shown in Supplementary Fig. 4a–d.

### DNA extraction: the first step

One gram of plant material (dried herbs, fresh aromatic herbs or ayurvedic powders) was manually crushed in liquid nitrogen using a mortar and pestle. Four ml of CTAB isolation buffer (2% Cetyl trimethylammonium bromide, 1.4 M NaCl, 20 mM EDTA, 100 mM Tris–HCl, pH 8.0 and 1% Polyvinylpyrrolidone 40, all from Sigma Aldrich) and 100 μl of proteinase k (from 20 mg/ml stock; Sigma Aldrich) was added into the fine powder and the mix was transferred into 50 ml polypropylene tube. Four ml of fresh skim milk (0.1% fat) was then added to the tube followed by incubation at either 37 °C for overnight (in case of freshly picked up aromatic herbs), or at 56 °C for overnight (in case of dried herbs and ayurvedic powders) on a rotating wheel in hybridization oven. Four ml of milliQ water was added in the untreated samples instead of skim milk to keep the volumes and concentration of detergent etc. constant for the treated and untreated samples. After the overnight incubation, 5 ml of chloroform/isoamyl alcohol (24:1 v/v) was added to each sample, gently mixed for 10 min and centrifuged at 10,000 rcf for 10 min at 4 °C. The supernatant was transferred to a new 15 ml polypropylene tube, 0.1 volume of 7.5 M ammonium acetate was added and mixed by inverting the tube 4–5 times. After mixing, two volume of 100% chilled ethanol was added to the sample to precipitate the DNA upon centrifugation at 10,000 rcf for 10 min at 4 °C. A big white or colored pellet of skim milk adsorbed DNA and other precipitated impurities is obtained at this stage, which is then washed with 70% ethanol, air dried and resuspended in 1 ml TE buffer (10 mM Tris, pH 8.0, 1 mM EDTA).

### DNA extraction: the second step

In the second step the precipitated skim milk adsorbed DNA was extracted to obtain the PCR quality DNA. For this, 1 ml CTAB buffer was added to the resuspended DNA solution from the above step, and incubated at room temperature for 5 min. The DNA was then extracted using routine phenol:chloroform procedure^18^. In the final step, the DNA was precipitated using 0.1 V of 3 M Sodium acetate (pH 5.2) and 0.6 V of chilled isopropanol. The pellet of pure DNA obtained was washed with 70% ethanol, air-dried and resuspended in 100 μl of TE buffer (10 mM Tris, pH 8.0, 0.1 mM EDTA) for downstream PCR amplification.

### PCR using animal specific universal primers mcb398 and mcb869

In order to assess the presence of contaminating DNA from the skim milk, the DNA obtained from the untreated and skim milk treated plant samples were amplified using animal specific universal primers mcb398 and mcb869 previously developed by us^19^ to generate the species-specific molecular signature (Supplementary Fig. 5). The, amplification was carried out in a 20 μl reaction volume containing 10 μl of 2 × Emrald Taq PCR mix (Takara, USA), 5 pM of each primer (mcb398 and mcb869), 7 μl miliQ water, and 1.0 μl template DNA (30–50 ng). The PCR conditions were: an initial denaturation at 95 °C for 5 min, followed by 35 cycles each of denaturation at 94 °C for 1 min, annealing at 51 °C for 1 min, and extension at 72 °C for 2 min. The extension step at the 35th cycle was held for 10 min. The 5 μl of PCR amplicons were loaded in 1% agarose gel to check the PCR success. The PCR products obtained were sequenced using Sanger sequencing in 3730Xl DNA Analyser (Thermo Fisher Scientific, USA) on both strands in duplicate and the sequence resolved were blasted against nr databases of NCBI using BLAST program and the ID of the species of contaminating DNA from skim milk was established using the procedure as described before^19^.

### Modified *matK* primers, PCR amplification and sequencing

The *matK* primers used in this study were modified from Jing et al.^20^ to give a robust amplification from a wide range of plant taxa. For this, more than 20 thousand available full and partial *matK* gene sequences were obtained from GenBank database of National Center for Biotechnology Information and aligned using ClustalX2 programme^21^ (data not shown). The aligned sequences were opened in BioEdit Sequence Alignment Editor V7.2.5^22^ and manually searched for the highly conserved regions to develop universal degenerate primers. The final sequence of the primers used in this study was—*matK*472F: 5′-CCCRTYCATCTGGAAATCTTGGTTC-3′ and *CCMB*-*matK*1248R: 5′-GCTRTRATAATGAGAAAGATTTCTGC-3′. The PCR reactions were conducted in a 20 μl mixture system containing 10 μl 2 × Emrald Taq PCR mix (Takara, USA), 5 pM of each primer, 7 μL miliQ water, and 1.0 μL template DNA (30–50 ng). The PCR conditions were: an initial denaturation at 95 °C for 5 min, followed by 35 cycles each of denaturation at 94 °C for 1 min, annealing at 54 °C for 1 min, and extension at 72 °C for 2 min. The final extension at 72 °C was held for 10 min. The PCR products obtained were sequenced using Sanger sequencing in 3730Xl DNA Analyser (Thermo Fisher Scientific, USA) on both strands in duplicate and the sequence resolved were blasted against nr databases of NCBI using BLAST program^23^ and the ID of the plant material was established using the procedure as described before^19^.

## Results and discussion

DNA isolation of herbal material can be a challenge due to inhibitors that result in sub-optimal DNA quality. This study takes a look at using skimmed-milk to enhancing the standard CTAB protocol in extracting DNA from herbal material. Here, we have shown that our modified CTAB extraction protocol using skim milk was able to generate quality DNA to successfully amplify PCR products. In addition, due to the issues surrounding the difficulty in the identification of herbal plant, we created our own primers to determine whether the herb materials used in the study have been correctly identified.

Skim milk is reported to prevent the degradation of DNA and its adsorption by soil colloids^17,24^. Skim milk was also found effective in extraction of DNA from recalcitrant soil that strongly adsorb DNA, indicating that it acts as the adsorbent competitors present in the crude lysates^25^. Taking the clue from above studies, we anticipated that the skim milk might also help in the extraction of pure DNA from dried aromatic herbs and herbal powders, where it is difficult to extract pure DNA due to the presence various secondary metabolites that inhibit the PCR reactions and hamper the downstream molecular applications. Our assumption was indeed found true, which was tested and validated in this study. The DNA was isolated from 12 randomly selected dried herbs (see “Methods”, Table 1, Fig. 1) with or without addition of skim milk in the first step of DNA isolation as described in Methods section. The DNA obtained from both sets of experiments was subjected to PCR amplification using modified *matK* primers developed in this study as described in Methods section. When the DNA was isolated from these dried herbs without addition of skim milk in the first step, only four samples produced desirable PCR product (Fig. 2b). The intensity of the PCR bands was low and multiple isolates from some of the samples did not produce reproducible results (Fig. 2b). However, skim milk treated samples consistently yielded PCR quality DNA from all of the 12 samples tested and a specific band of expected size and high intensity was obtained (Fig. 2d). Contamination with exogenous DNA from skim milk was negligible as the genomic DNA was not detected in skim milk treated internal blank controls (data not shown), and subsequent PCR of *matK* gene did not produce any detectable band in internal negative control (Fig. 2d). However, the presence of animal specific exogenous DNA in skim milk treated herb DNA samples was confirmed by the PCR amplification of these samples using animal specific universal primers (mcb398 and mcb869)^19^ (Supplementary Fig. 5). A band of specific size (472 bp) was obtained in skim milk treated dried herbs samples; however, no band was detected in the DNA from untreated dried herbs using these animal specific universal primers (Supplementary Fig. 5). The species origin of the contaminating DNA from skim milk was confirmed to that of *Bos taurus* (cow) as summarized in Supplementary Tables 1 and 2. This experiment confirmed that the presence of animal origin exogenous DNA from skim milk did not affect the *matK* based PCR amplication of plant DNA for downstream analysis.

The applicability of this method of DNA isolation from herbs was further assessed and validated with an additional 35 randomly picked up fresh aromatic herb samples collected from countryside land near Hyderabad, India (Supplementary Fig. 1, Supplementary Table 3), six additional dried herbs (total 18, Table 2) obtained from online herbal marketplace (Fig. 1, Table 2), and five herbal powders obtained from a traditional practitioner of Ayurveda (Supplementary Fig. 2, Supplementary Table 4). For all the samples tested, our method successfully yielded PCR quality DNA, which was subsequently amplified and sequenced using *matK* primers confirming that the DNA obtained was good enough for the downstream PCR applications (All nucleotide Sequences deposited in NCBI database as accession Nos MN006706 to MN006768). The *matK* based DNA typing^9^ was also able to establish the identity of the fresh aromatic herbs (Supplementary Fig. 1) either at the species level or at the genus level with high BLAST scores as shown in Supplementary Table 3.

Furthermore, we also checked the authenticity of the dried herbs and herbal powders included in this study using *matK* based DNA typing. It was found that of the 18 dried herbs tested, a total of 15 were identified as the same herb as it was mentioned on its label by online seller; however, plant species origin of one of the dried samples, which was sold to us in online marketplace with the spurious name ‘Dashmool Bharad’ (Sample—H7), was found to be the that of *Tribulus sp.* (Fig. 1, Table 2). Similarly, the identity of two other herbal samples (Samples—H12 and H21 in Fig. 1) was found spurious (see footnotes in Table 2) either due to mislabeling or fraudulently replacement, indicating the incidences of malpractices in online herbal marketplace.

Furthermore, three of the five ayurvedic powders were also identified as that of the same herb’s origin as labeled by local ayurvedic practitioner; however, the identity of two of the samples was found spurious (Supplementary Fig. 2, Supplementary Table 4). These findings were confirmed by careful examination of high BLAST scores of the IDs of each specimen tested with multiple specimen vouchers entries in ncbi database, and also by inclusion of reference samples for some of the specimen tested in this study (Supplementary Fig. 3, Supplementary Table 5). These results provided initial DNA based evidence of the instances of malpractices in online herbal marketplace, these findings; however, need to be confirmed by comparing with a high fidelity reference database of authentic, dried medicinal herbs being used in various systems of plants based medicines around the world, which still needed to be done. Further investigation on such mislabeling or fraudulently replacement of dried herbs in online herbal marketplace will be presented elsewhere in detail as this is beyond the scope of this work.

This study provides a novel, validated method of DNA isolation from dried aromatic herbs and herbal powders, which could be used as an economic alternative to successfully extract PCR quality DNA from such plant materials. The presented milk protocol was an “improved” protocol compared to the standard CTAB protocol of DNA isolation as shown in this study. Nevertheless we have not compared this protocol with alternative DNA extraction protocols and commercial kits; it would be interesting to see whether it outperforms even the alternative extraction protocols and available commercial kits for plant DNA extraction. In its current format, our method seems to be labor-intensive in terms of time relative to the commercial kits, but it is possible that inclusion of novel step of pretreatment of plant samples with skim milk may even enhance the success rates and efficiency of such kits in future.

This study further present a set of robust *matK* primers which could be used as plant barcoding resource in future studies. While validating the utility of our method of plant DNA isolation and typing in a brief molecular survey of randomly selected dried herbs obtained from online marketplace and herbal powders obtained from local ayurvedic practitioner, we also noticed the instances of malpractices or mislabeling of dried herbs and herbal powders, which are reported in this study and further seek an urgent attention. This also indicates the urgent need for a global effort to develop a high fidelity reference database of authentic, dried medicinal herbs which are being used in various systems of plants based medicines around the world.

## Supplementary information

Supplementary information.

## Data Availability

All sequencing data that support the findings of this study have been deposited in the National Center for Biotechnology Information “GenBank” (https://www.ncbi.nlm.nih.gov/) with the accession codes MN006706 to MN006768. All other data supporting the findings of this study are available within the paper and its supplementary information files.
