# Supplementary material for: A novel nucleic acid extraction method from aromatic herbs and dried herbal powders using cow skim milk
Source: Sci Rep. 2020 Jul 13;10:11513. doi: 10.1038/s41598-020-68467-4 (PMC7359320; doi:10.1038/s41598-020-68467-4)
Supplement: Supplementary file 1 — Supplementary information. [file 41598_2020_68467_MOESM1_ESM.docx]

**Supplementary information**

**Title: A novel nucleic acid extraction method from aromatic herbs and dried herbal powders using cow skim milk**

Authors: Sunil Kumar Verma* & Nabanita Biswas

Affiliation:

CSIR - Centre for Cellular and Molecular Biology, Uppal Road, Hyderabad 500 007, India

*Author for Correspondence

Sunil Kumar Verma

S212, CSIR – Centre for Cellular and Molecular Biology, Uppal Road, Hyderabad 500 007, India

Email: [sunilverma@ccmb.res.in](mailto:sunilverma@ccmb.res.in)

**Supplementary Table 1:**

The partial cytochrome b sequences delineated from the DNA samples analysed using animal specific universal primers ‘mcb398 and mcb869’ as shown in supplementary figure 5.

| **S.No.** | **Reference Code** | **Sequence (5’ -3’)** |
| --- | --- | --- |
| 1 | *Bos Taurus (+ve control)* | >Bos_taurus  AGGAGCAACAGTCATCACCAACCTCTTATCAGCAATCCCATACATCGGCACAAATTTAGTCGAATGAATCTGAGGCGGA  TTCTCAGTAGACAAAGCAACCCTTACCCGATTCTTCGCTTTCCATTTTATCCTTCCATTTATCATCATAGCAATTGCCA  TAGTCCACCTATTATTCCTCCACGAAACAGGCTCCAACAATCCAACAGGAATCTCCTCAGACGTAGACAAAATCCCATT  CCACCCCTACTATACCATTAAGGACATCTTAGGGGCCCTCTTACTAATTCTAGCTCTAATACTACTAGTACTATTCGCA  CCCGACCTCCTCGGAGACCCAGATAACTACACCCCGGCCAATCCACTCAACACACCTCCTCACATCAAACCCGAATGAT  ACTTCTTATTTGCATACGCAATCTTA |
| 2 | Human *(+ve control)* | >Human  AGGGGCCACAGTAATTACAAACTTACTATCCGCCATCCCATACATTGGGACAGACCTAGTTCAATGAATCTGAGGAGGC  TACTCAGTAGACAGTCCCACCCTCACACGATTCTTTACCTTTCACTTCATCTTGCCCTTCATTATTGCAGCCCTAGCAG  CACTCCACCTCCTATTCTTGCACGAAACGGGATCAAACAACCCCCTAGGAATCACCTCCCATTCCGATAAAATCACCTT  CCACCCTTACTACACAATCAAAGACGCCCTCGGCTTACTTCTCTTCCTTCTCTCCTTAATGACATTAACACTATTCTCA  CCAGACCTCCTAGGCGACCCAGACAATTATACCCTAGCCAACCCCTTAAACACCCCTCCCCACATCAAGCCCGAATGAT  ATTTCCTATTCGCCTACACAATTCTC |
| 3 | H8 | >H8  AGGAGCAACAGTCATCACCAACCTCTTATCAGCAATCCCATACATCGGCACAAATTTAGTCGAATGAATCTGAGGCGGA  TTCTCAGTAGACAAAGCAACCCTTACCCGATTCTTCGCTTTCCATTTTATCCTTCCATTTATCATCATAGCAATTGCCA  TAGTCCACCTATTATTCCTCCACGAAACAGGCTCCAACAATCCAACAGGAATCTCCTCAGACGTAGACAAAATCCCATT  CCACCCCTACTATACCATTAAGGACATCTTAGGGGCCCTCTTACTAATTCTAGCTCTAATACTACTAGTACTATTCGCA  CCCGACCTCCTCGGAGACCCAGATAACTACACCCCGGCCAATCCACTCAACACACCTCCTCACATCAAACCCGAATGAT  ACTTCTTATTTGCATACGCAATCTTA |
| 4 | H14 | >H14  AATGAATCTGAGGCGGATTCTCAGTAGACAAAGCAACCCTTACCCGATTCTTCGCTTTCCATTTTATCCTTCCATTTAT  CATCATAGCAATTGCCATAGTCCACCTATTATTCCTCCACGAAACAGGCTCCAACAATCCAACAGGAATCTCCTCAGAC  GTAGACAAAATCCCATTCCACCCCTACTATACCATTAAGGACATCTTAGGGGCCCTCTTACTAATTCTAGCTCTAATAC  TACTAGTACTATTCGCACCCGACCTCCTCGGAGACCCAGATAACTACACCCCGGCCAATCCACTCAACACACCTCCTCA  CATCA |
| 5 | H19 | Sample lost during processing |
| 6 | H24 | >H24  AGGAGCAACAGTCATCACCAACCTCTTATCAGCAATCCCATACATCGGCACAAATTTAGTCGAATGAATCTGAGGCGGA  TTCTCAGTAGACAAAGCAACCCTTACCCGATTCTTCGCTTTCCATTTTATCCTTCCATTTATCATCATAGCAATTGCCA  TAGTCCACCTATTATTCCTCCACGAAACAGGCTCCAACAATCCAACAGGAATCTCCTCAGACGTAGACAAAATCCCATT  CCACCCCTACTATACCATTAAGGACATCTTAGGGGCCCTCTTACTAATTCTAGCTCTAATACTACTAGTACTATTCGCA  CCCGACCTCCTCGGAGACCCAGATAACTACACCCCGGCCAATCCACTCAACACACCTCCTCACATCAAACCCGAATGAT  ACTTCTTATTTGCATACGCAATCTTA |
| 7 | H49 | >H49  AGGAGCAACAGTCATCACCAACCTCTTATCAGCAATCCCATACATCGGCACAAATTTAGTCGAATGAATCTGAGGCGGA  TTCTCAGTAGACAAAGCAACCCTTACCCGATTCTTCGCTTTCCATTTTATCCTTCCATTTATCATCATAGCAATTGCCA  TAGTCCACCTATTATTCCTCCACGAAACAGGCTCCAACAATCCAACAGGAATCTCCTCAGACGTAGACAAAATCCCATT  CCACCCCTACTATACCATTAAGGACATCTTAGGGGCCCTCTTACTAATTCTAGCTCTAATACTACTAGTACTATTCGCA  CCCGACCTCCTCGGAGACCCAGATAACTACACCCCGGCCAATCCACTCAACACACCTCCTCACATCAAACCCGAATGAT  ACTTCTTATTTGCATACGCAATCTTA |
| 8 | H54 | >H54  AGGAGCAACAGTCATCACCAACCTCTTATCAGCAATCCCATACATCGGCACAAATTTAGTCGAATGAATCTGAGGCGGA  TTCTCAGTAGACAAAGCAACCCTTACCCGATTCTTCGCTTTCCATTTTATCCTTCCATTTATCATCATAGCAATTGCCA  TAGTCCACCTATTATTCCTCCACGAAACAGGCTCCAACAATCCAACAGGAATCTCCTCAGACGTAGACAAAATCCCATT  CCACCCCTACTATACCATTAAGGACATCTTAGGGGCCCTCTTACTAATTCTAGCTCTAATACTACTAGTACTATTCGCA  CCCGACCTCCTCGGAGACCCAGATAACTACACCCCGGCCAATCCACTCAACACACCTCCTCACATCAAACCCGAATGAT  ACTTCTTATTTGCATACGCAATCTTA |

**Supplementary Table 2:**

Species identity of the +ve control DNA samples and the remnant of exogenous animal DNA present in the skim milk treated herbs samples as revealed by animal specific universal primers mcb398 and mcb869 (Verma and Singh

| **S.N.** | **Reference Code *** | **Highest Bits** | **Query Cover %** | **BLAST E Value** | **% Nucleotide Similarity** | **NCBI Accession No.^†^** | **Identity Revealed As** |
| --- | --- | --- | --- | --- | --- | --- | --- |
| 1 | *Bos Taurus (+ve control)* | 778 | 100 | 0.0 | 100 | MH714784.1 | *Bos taurus* |
| 3 | Human *(+ve control)* | 778 | 100 | 0.0 | 100 | MH550165.1 | *Homo sapiens* |
| 3 | H8 | 778 | 100 | 0.0 | 100 | MH714784.1 | *Bos taurus* |
| 4 | H14 | 593 | 100 | 1e-165 | 100 | MH714784.1 | *Bos taurus* |
| 5 | H19^‡^ | - | | | | | |
| 6 | H24 | 778 | 100 | 0.0 | 100 | MH714784.1 | *Bos taurus* |
| 7 | H49 | 778 | 100 | 0.0 | 100 | MH714784.1 | *Bos taurus* |
| 8 | H54 | 778 | 100 | 0.0 | 100 | MH714784.1 | *Bos taurus* |

* All the sequences delineated from these samples using universal primers mcb398 and mcb869 are given in Supplementary Table 1

**^†^** NCBI Accession No. of the best BLAST hit with the corresponding sequences in NCBI database

^‡^This sample was lost during sequence processing

**Supplementary Table 3:**

Various Fresh Aromatic Herbs included in this study to validate the DNA extraction procedure and the success of downstream molecular analysis

| **S.N.** | **Sample Code** | **Herb Seq Voucher Code** | **NCBI Accession No*** | **Highest Bits** | **Query Cover %** | **BLAST E Value** | **% Nucleotide Similarity** | **NCBI Accession No.**** | **Herb Identity Revealed As** |
| --- | --- | --- | --- | --- | --- | --- | --- | --- | --- |
| 1 | WB5 | CCMB:27-100:WB5 | MN006706 | 1242 | 100 | 0.0 | 100 | MH767935.1 | *Sida acuta* |
| 2 | WB6 | CCMB:27-92:WB6 | MN006707 | 1343 | 100 | 0.0 | 100 | EU371806.1 | *Cleome viscosa* |
| 3 | WB13 | CCMB:27-101:WB13 | MN006708 | 1354 | 100 | 0.0 | 99.86 | AM887524.1 | *Gomphrena ferruginea* |
| 4 | WB14 | CCMB:27-102:WB14 | MN006709 | 1349 | 100 | 0.0 | 99.86 | KT966997.1 | *Sida rhombifolia* |
| 5 | WB15 | CCMB:27-103:WB15 | MN006710 | 1315 | 100 | 0.0 | 98.40 | MH621641.1 | *Indigofera miniata* |
| 6 | WB16 | CCMB:27-104:WB16 | MN006711 | 1371 | 100 | 0.0 | 100 | KY952353.1 | *Boerhavia repens* |
| 7 | WB17 | CCMB:27-105:WB17 | MN006712 | 1382 | 100 | 0.0 | 100 | MH621608.1 | *Cyanthillium cinereum* |
| 8 | WB31 | CCMB:28-113:WB31 | MN006713 | 1254 | 100 | 0.0 | 100 | MH017987.1 | *Parthenium hysterophorus* |
| 9 | SJ2 | CCMB:26-84:SJ2 | MN006714 | 1304 | 96 | 0.0 | 99.31 | MH748946.1 | *Crossandra infundibuliformis* |
| 10 | SJ3 | CCMB:26-85:SJ3 | MN006715 | 1155 | 100 | 0.0 | 99.68 | MF349897.1 | *Andrographis paniculata* |
| 11 | SJ4 | CCMB:26-86:SJ4.1 | MN006716 | 1192 | 100 | 0.0 | 100 | LC102228.1 | *Citrus sp.* *CCMB SJ4* |
| 12 | WB1 | CCMB:27-96:WB1 | MN006717 | 1365 | 100 | 0.0 | 99.87 | FJ235271.1 | *Phyllanthus sp.* *CCMB WB1* |
| 13 | WB2 | CCMB:27-97:WB2.1 | MN006718 | 1371 | 100 | 0.0 | 100 | MK125099.1 | *Trianthema portulacastrum* |
| 14 | WB7 | CCMB:27-91:WB7 | MN006719 | 1354 | 100 | 0.0 | 100 | KY645505.1 | *Abutilon abutiloides* |
| 15 | WB20 | CCMB:28-106:WB20 | MN006720 | 418 | 99 | 4e-113 | 100 | MH552308.1 | *Euphorbia hirta* |
| 16 | WB21 | CCMB:28-107:WB21 | MN006721 | 1218 | 100 | 0.0 | 100 | MH767933.1 | *Malvastrum coromandelianum* |
| 17 | WB22 | CCMB:28-108:WB22 | MN006722 | 758 | 95 | 0.0 | 100 | LT992585.1 | *Gomphrena celosioides* |
| 18 | WB23 | CCMB:28-109:WB23 | MN006723 | 621 | 84 | 1e-173 | 100 | MK397889.1 | *Portulaca oleracea* |
| 19 | WB32 | CCMB:28-114:WB32 | MN006724 | 1280 | 100 | 0.0 | 100 | MH767977.1 | *Oxalis corniculata* |
| 20 | WB33 | CCMB:29-116:WB33 | MN006725 | 1277 | 100 | 0.0 | 100 | MG836507.1 | *Amaranthus sp.* *CCMB WB33* |
| 21 | WB34 | CCMB:29-117:WB34 | MN006726 | 1306 | 100 | 0.0 | 100 | MH767970.1 | *Boerhavia sp.* *CCMB WB34* |
| 22 | SV2 | CCMB:26-88:SV2.2 | MN006727 | 1214 | 100 | 0.0 | 100 | MF807938.1 | *Passiflora edulis* |
| 23 | SV4 | CCMB:26-89:SV4 | MN006728 | 1092 | 100 | 0.0 | 100 | AM920611.1 | *Syngonium auritum* |
| 24 | SV5 | CCMB:29-124:SV5 | MN006729 | 1349 | 100 | 0.0 | 100 | GU135034.1 | *Syngonium podophyllum* |
| 25 | SV8 | CCMB:29-125:SV8 | MN006730 | 1341 | 100 | 0.0 | 99.86 | KC019406.1 | *Euphorbia magnifica* |
| 26 | SV9 | CCMB:29-126:SV9.2 | MN006731 | 1312 | 100 | 0.0 | 100 | KJ150223.1 | *Jatropha podagrica* |
| 27 | SV10 | CCMB:29-127:SV10.1 | MN006732 | 1373 | 100 | 0.0 | 99.87 | KX783670.1 | *Duranta sp.* *CCMB SV10* |
| 28 | WB3 | CCMB:27-98:WB3 | MN006733 | 1332 | 100 | 0.0 | 99.72 | EU371806.1 | *Cleome viscosa* |
| 29 | WB10 | CCMB:27-95:WB10 | MN006734 | 1206 | 100 | 0.0 | 100 | KX783763.1 | *Polyscias sp.* *CCMB WB10* |
| 30 | WB24 | CCMB:28-110:WB24 | MN006735 | 1243 | 100 | 0.0 | 100 | MH767840.1 | *Acalypha lanceolata* |
| 31 | WB25 | CCMB:28-111:WB25 | MN006736 | 1258 | 100 | 0.0 | 100 | LK021437.1 | *Euphorbia sp.* *CCMB WB25* |
| 32 | WB26 | CCMB:28-112:WB26 | MN006737 | 1271 | 100 | 0.0 | 100 | MH551782.1 | *Euphorbia prostrata* |
| 33 | WB37 | CCMB:29-119:WB37 | MN006738 | 1286 | 100 | 0.0 | 100 | MF694861.1 | *Lantana camara* |
| 34 | WB39 | CCMB:29-120:WB39 | MN006739 | 1286 | 100 | 0.0 | 100 | MF694842.1 | *Conyza bonariensis* |
| 35 | WB40 | CCMB:29-121:WB40 | MN006740 | 1310 | 100 | 0.0 | 100 | KX147312.1 | *Coccinia grandis* |

Photographs of each of these aromatic herbs are provided in Supplementary Figure 1.

* NCBI Accession No. of the novel sequences generated in this study.

****** NCBI Accession No. of the best BLAST hit with the corresponding sequences in NCBI database

**Supplementary Table 4:**

List of Ayurvedic powders included in this study to validate the DNA extraction procedure developed and the success of downstream molecular analysis

| **S.N.** | **Sample Code** | **Ayurvedic Powder Name as on Label / Voucher Code** | ***NCBI Accession No** | **Highest Bits** | **Query Cover %** | **BLAST E Value** | **% Nucleotide Similarity** | **NCBI Accession No. **** | **Herb Identity Revealed As** |
| --- | --- | --- | --- | --- | --- | --- | --- | --- | --- |
| 1 | PW4 | Bael - *Aegle marmelos*  (CCMB:20-34:PW4) | MN006759 | 518 | 94 | 1e-142 | 97.67 | AB762358.1 | *Aegle marmelos* |
| 2 | PW6 | Aswagandha – *Withania sp.*  (CCMB:20-30:PW6) | MN006760 | 1260 | 100 | 0.0 | 99.85 | MF694890.1 | *Withania sp.* *CCMB PW6* |
| 3 | PW11 | Sanaya – *Senna sp.*  (CCMB:20-31:PW11) | MN006761 | 1227 | 100 | 0.0 | 100 | MH558313.1 | *Senna sp.* *CCMB PW11* |
| 4 | ^†^ PW15 | Vidhara - *Argyreia nervosa*  (CCMB:20-32:PW15) | MN006762 | 785 | 100 | 0.0 | 97.55 | KX119398.1 | *Mucuna sp.* *CCMB PW15* |
| 5 | ^†^ PW24 | Bakuchi - *Psoralea corylifolia*  (CCMB:20-33:PW24) | MN006763 | 534 | 100 | 6e-148 | 99.66 | MF651949.1 | *Sapindus sp.* *CCMB PW24* |

Photographs of each of these ayurvedic powders are provided in Supplementary Figure 2.

* NCBI Accession Nos. of the novel sequences generated in this study.

****** NCBI Accession No. of the best BLAST hit with the corresponding sequences in NCBI database

^†^ Identity of these ayurvedic powders was found to be spurious either due to mislabeling or fraudulently replacement, indicating the incidences of malpractices or unintentional negligence.

**Supplementary Table 5:**

List of reference samples and their IDs as confirmed by molecular analysis

| **S.N.** | **Reference Code** | **Reference Herb ID** | ***NCBI Accession No** | **Highest Bits** | **Query Cover %** | **BLAST E Value** | **% Nucleotide Similarity** | **NCBI Accession No. **** | **Herb Identity Revealed As** |
| --- | --- | --- | --- | --- | --- | --- | --- | --- | --- |
| 1 | WB35 | *Tribulus sp.*  *(*CCMB:29-118:WB35) | MN006764 | 1290 | 100 | 0.0 | 99.86 | MF694887.1 | *Tribulus sp.* *CCMB WB35* |
| 2 | CH73 | *Adhatoda vasica / Justicia adhatoda*  *(CCMB:71-230:CH73)* | MN006765 | 1382 | 100 | 0.0 | 100 | KX783699.1 | *^1^Justicia adhatoda/ Adhatoda vasica* |
| 3 | CH10 | *Piper longum*  *(CCMB:58-179:CH10)* | MN006766 | 1365 | 100 | 0.0 | 99.87 | MH287271.1 | *Piper longum* |
| 4 | SV3/J | *Psoralea corylifolia*  *(CCMB:48-162:SV3J)* | MN006767 | 1236 | 100 | 0.0 | 100 | MK069582.1 | *^2^Cullen corylifolium/* *Psoralea corylifolia* |
| 5 | WB4 | *Butea monosperma*  *(CCMB:27-99:WB4)* | MN006768 | 1387 | 100 | 0.0 | 100 | JN008175.1 | *Butea monosperma* |

Photographs of each of these reference herbs are provided in Supplementary Figure 3.

* NCBI Accession Nos. of the novel sequences generated in this study.

****** NCBI Accession No. of the best BLAST hit with the corresponding sequences in NCBI database

^1, 2^ *Adhatoda vasica* and *Justicia adhatod* are synonyms. Similarly genus *Psoralea* and *Cullen* are also synonyms.

**Fig. 1**


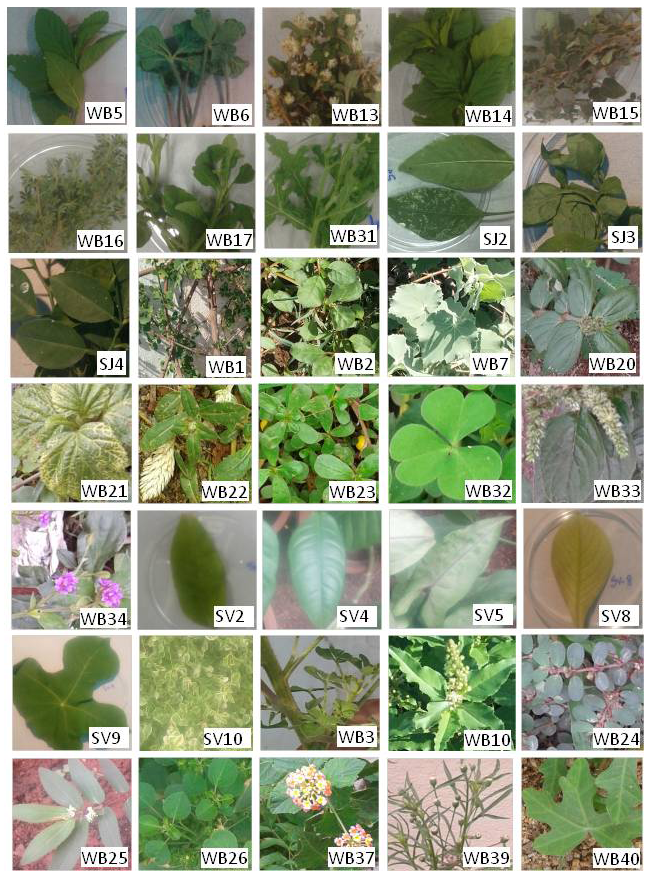


**Supplementary Figure 1:** Fresh Aromatic herbs included in this study. Refer to Supplementary Table 3 for the details of each herb.

**Fig. 2**


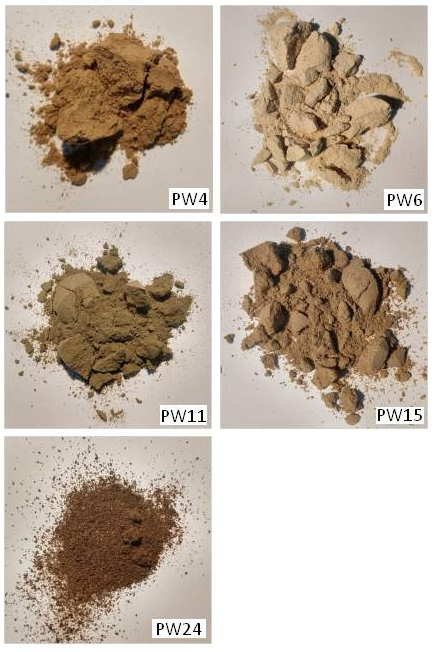


**Supplementary Figure 2:** Ayurvedic powders included in this study. Refer to Supplementary Table 4 for the details.

**Fig. 3**


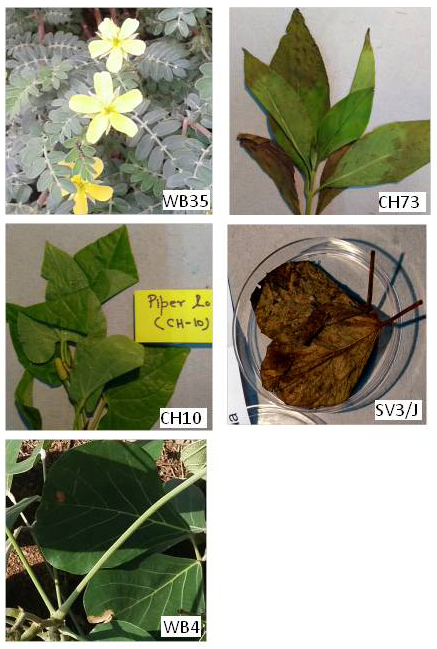


**Supplementary Figure 3:** Reference herb samples used in this study. Refer to Supplementary Table 5 for the details.

**Supplementary Figure 4a:**

The Uncropped, multiple original exposure of the full length agarose gel displayed in panel ‘a’ of ‘Figure 2’ in main text

**Supplementary Figure 4b:**

The Uncropped, multiple original exposure of the full length agarose gel displayed in panel ‘b’ of ‘Figure 2’ in main text

**Supplementary Figure 4c:**

The Uncropped, multiple original exposure of the full length agarose gel displayed in panel ‘c’ of ‘Figure 2’ in main text

**Supplementary Figure 4d:**

The Uncropped, multiple original exposure of the full length agarose gel displayed in panel ‘d’ of ‘Figure 2’ in main text

Figure 5: **Agarose gel showing PCR amplifications of DNA obtained from untreated and skim milk treated plant samples ( as marked) using animal specific universal primers (mcb398 and mcb869)** ^25^**.**

The lanes marked as ‘+ve’ are the PCR amplicons obtained from known DNA from *Bos taurus* (Cow) and *Homo sapiens* (Human) source. The lanes marked as ‘–ve’ are the ‘negative’ controls for the PCR reactions. The molecular weight markers (Lane M) used are Next Gen 100-1500 DNA Ladder (Cat. No. 516469). The DNA from known animal species source (Cow and Human as indicated) yielded the band of specific size (472bp) as expected. The DNA from untreated herbs as indicated did not amplify using these animal specific universal primers; however, the DNA obtained from skim milk treated herbs gave a band of specific size, indicating the presence of remnant of animal DNA in skim milk treated herbs samples. All the PCR amplicons obtained from this experiment (except sample H19, which was lost during processing) were sequenced using the universal primers mcb398 and mcb869 and the species Identity of the contaminating DNA was established using standard BLAST procedure as described by ^25^ (see Supplementary Table 1 and Supplementary Table 2).
